# Ewing Sarcoma of the Scapula: A Rare Case of a 10-Year-Old Child With a Two-Year Follow-Up

**DOI:** 10.7759/cureus.72376

**Published:** 2024-10-25

**Authors:** Shoog F Alfadhel, Osama AlShaya

**Affiliations:** 1 Reconstructive Orthopedic Department, King Fahad Medical City, Riyadh, SAU

**Keywords:** adolescent, ewing sarcoma, radiotherapy (rt), rare osteosarcoma, scapula tumors

## Abstract

Ewing sarcoma is a very common type of malignant bone tumor among children and adolescents that most frequently develops in long bones of the body and extremities; however, it can also affect other bones such as the skull and scapula in rare cases. In Ewing sarcoma, the most common sites of metastasis are the lungs and bones, which indicates a late stage of the disease and is considered a poor prognostic factor. In this paper, we report the case of a 10-year-old boy presenting with a painless swelling on his left shoulder that was not associated with other local or systemic symptoms, and with no neurological manifestations. Initially, Ewing sarcoma of the scapula was suspected after radiological studies, including ultrasonography, X-ray, computed tomography, and bone scintigraphy, which was eventually confirmed by histopathology and immunohistochemistry showing the typical findings of Ewing sarcoma. Ewing sarcoma of the scapula is a rare condition, with only 18 cases reported to the best of our knowledge.

## Introduction

Ewing sarcoma, named after James Ewing, is a type of malignant bone tumor that is more prevalent among children and young adults aged 10-20 years [1,2]. It is the third most common primary sarcoma of the bone, accounting for approximately 10% of all primary malignant bone tumors [3]. In 85% of Ewing sarcoma cases, translocation is usually present, resulting in the formation of EWS-FLI1 fusion gene products, which is a known cause of this cancer type [4]. Ewing sarcoma is known to mainly arise from the metaphysis or diaphysis of long bones such as the tibia and femur [5], whereas it rarely develops in the vertebra, skull, or scapula [1]. In fact, tumors rarely develop in the scapula, as reported in a retrospective study done in Mexico including 566 patients with bone tumors in which the scapula was affected in only 1.6% of cases, whereas the femur was found to be the most commonly affected bone, accounting for 39.9% of cases [6]. Lungs and other bones are the most common sites of metastasis [7].

The most common symptoms of Ewing sarcoma are pain at the site of the lesion or swelling, which is sometimes delayed, unfortunately manifesting at a later stage of the disease [8]. Radiographic imaging modalities such as computed tomography (CT) and magnetic resonance imaging (MRI) are essential for diagnosis, along with histopathological confirmation of the disease [9]. Positron emission tomography-computed tomography is also helpful to determine the presence of distant metastasis, providing guidance for planning the management of the disease and the goals of treatment [9]. Over time, the prognosis of Ewing sarcoma has significantly improved owing to ongoing advances in chemotherapy [10]. Generally, a multidisciplinary approach including surgery, chemotherapy, radiotherapy, and multiple other specialties is crucial for successful management of these tumors, especially those arising from bones in which tumors rarely develop, such as the scapula [9].

Ewing sarcoma is a rare occurrence in the scapula, which itself is a bone from which tumors rarely develop, with only approximately 18 cases reported in PubMed. This article reports the case of a 10-year-old boy presenting with painless swelling of the left shoulder, which was eventually diagnosed as Ewing sarcoma of the scapula.

## Case presentation

A 10-year-old boy with no significant medical history presented to our emergency room with painless swelling of the left shoulder after sustaining direct trauma after being pushed by his brother while they were playing 10 days prior. The swelling was not associated with any other symptoms such as pain, fever, or limited range of motion. On physical examination, he was vitally stable with unremarkable examination aside from the left-shoulder swelling, which was found to be a well-defined, slightly mobile, hard, subcutaneous mass located over the left scapula measuring approximately 7 cm x 10 cm. There was no associated tenderness, open wounds, or sinuses or signs of infection. His range of motion was fully intact and completely unaffected.

After preforming laboratory studies, the patient’s complete blood count (CBC) was unremarkable, and his chemistry and bone panel were only remarkable for mild hypocalcemia (1.99 mmol/L) and high lactate dehydrogenase (LDH; 537 U/L). His coagulation profile was slightly elevated, with a prothrombin time of 12.6 s and an activated partial thromboplastin time of 42.2 s.

**Figure 1:**
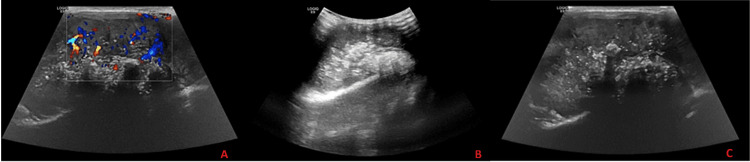
Ultrasonography images of the left scapula showing a heterogenous mass in the back of the left shoulder measuring 9.7 cm x 5 cm with (A) internal vascularity, (B) scattered calcifications, and (C) calcification

**Figure 2:**
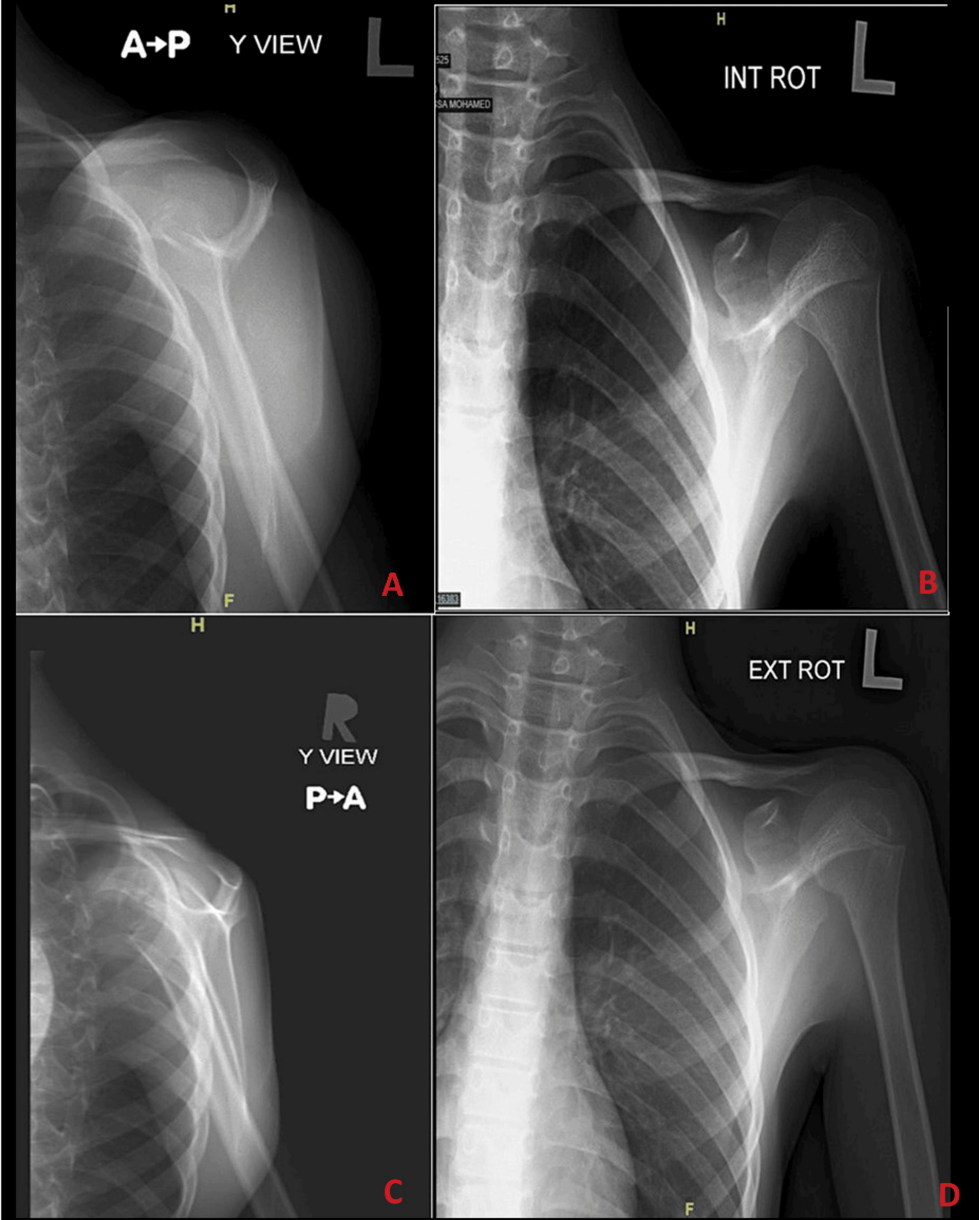
X-rays of the left shoulder showing significant soft-tissue thickening and swelling around the left scapula, with preserved alignment and joint spaces: (A) anteroposterior view of left shoulder; (B) internal rotation view of left shoulder; (C) Y-view of left shoulder; and (D) external rotation view of left shoulder

**Figure 3:**
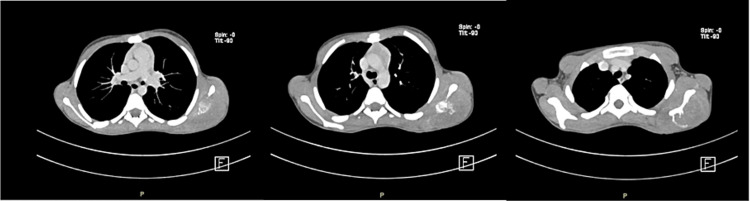
Computed tomography scan of the chest showing a large heterogenous destructive mass in the left scapula with calcified matrix, a large soft-tissue mass, and no evidence of lung metastasis

**Figure 4:**
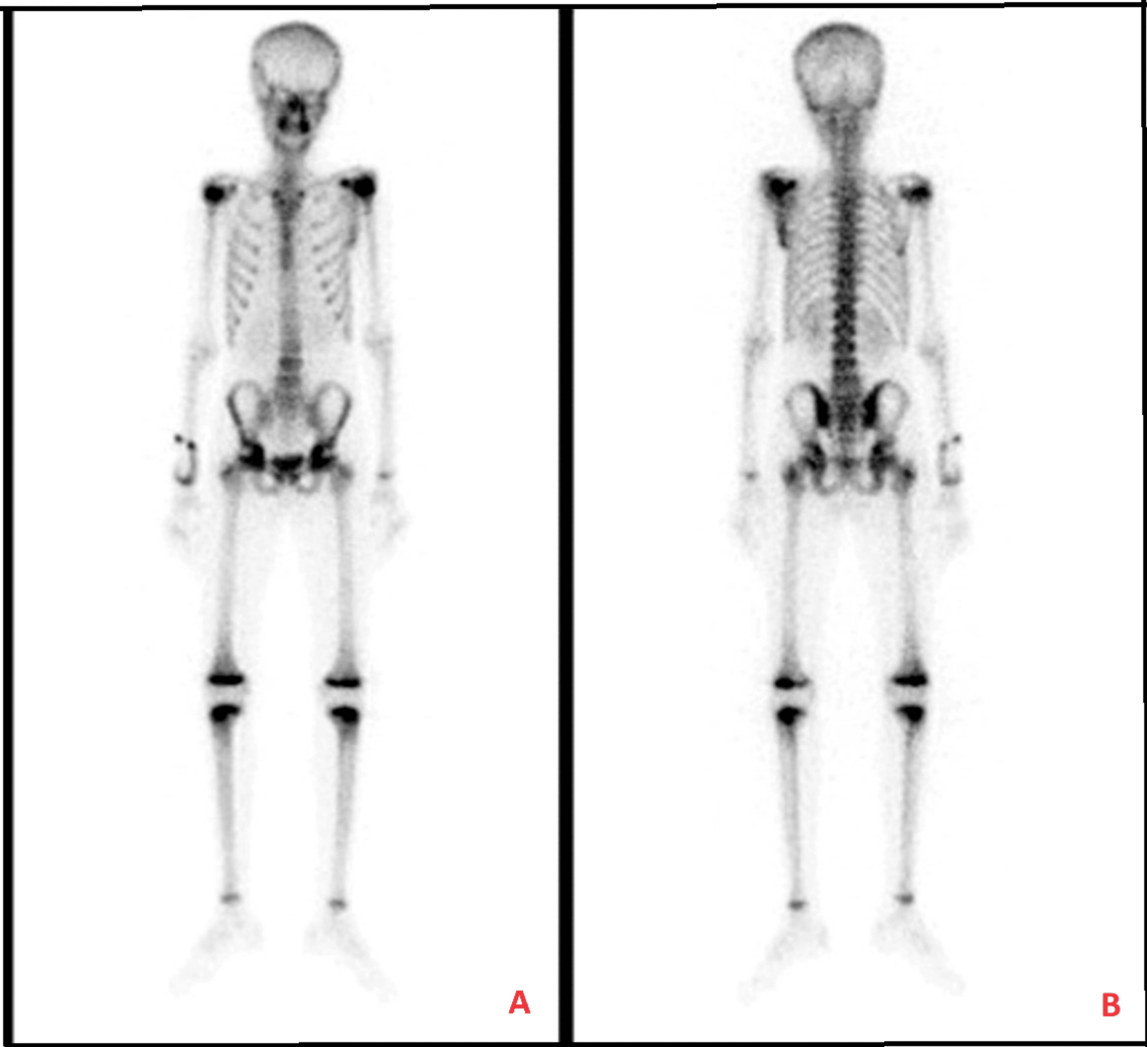
Bone scintigraphy showing a solitary-avid left scapular lesion with no evidence of distant osteoblastic bone metastasis: (A) anterior view and (B) posterior view

Initially, shoulder ultrasonography (US) showed a heterogenous mass in the back of the left shoulder measuring 9.7 cm x 5 cm with scattered calcifications and internal vascularity (Figure 1). A shoulder X-ray showed significant soft-tissue thickening and swelling noted around the left scapula and preserved alignment and joint spaces (Figure 2). A subsequent CT chest-abdomen-pelvis (CAP) scan showed a large, heterogenous, destructive mass in the left scapula with a calcified matrix measuring 6.3 cm x 5.6 cm x 10.9 cm, a large soft-tissue mass, and no evidence of lung metastasis or other abnormalities in the abdomen or pelvis (Figure 3). A Tc-99m methylene diphosphonate (MDP) bone scintigraphy study was carried out, which showed a solitary MDP-avid left scapular lesion with no evidence of distant osteoblastic bone metastasis (Figure 4).

After a complete radiological evaluation, Ewing sarcoma of the scapula was suspected, which required histological confirmation with biopsy. A biopsy of the left-shoulder soft-tissue mass was taken, which showed a tumor comprising small, round, blue cells favoring diagnosis of Ewing sarcoma. An immunohistochemistry of the tumor cells showed that they were positive for CD99 and synaptophysin but negative for S100, TdT, CD20, desmin, SMA, CK, chromogranin, CD3, and EMA.

**Figure 5:**
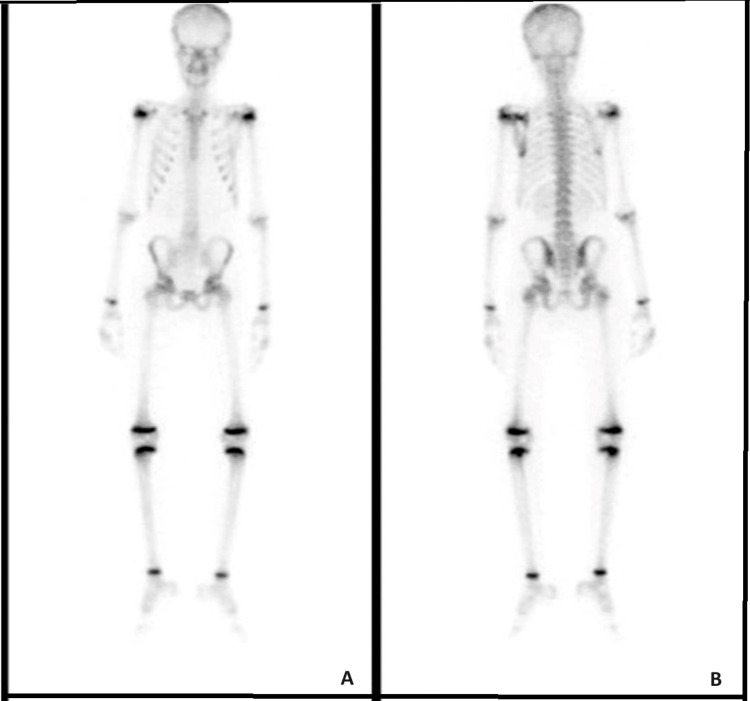
Bone scintigraphy taken after completion of chemotherapy showing mild interval improvement of known left scapula Ewing sarcoma: (A) anterior view and (B) posterior view

**Figure 6:**
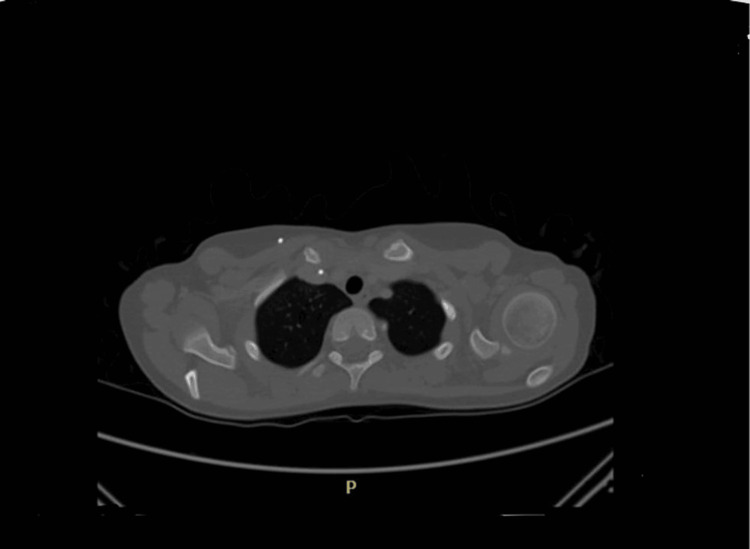
Computed tomography scan of the left scapula post-chemotherapy showing significant interval regression in the size and appearance of the left scapular soft-tissue mass lesion

**Figure 7:**
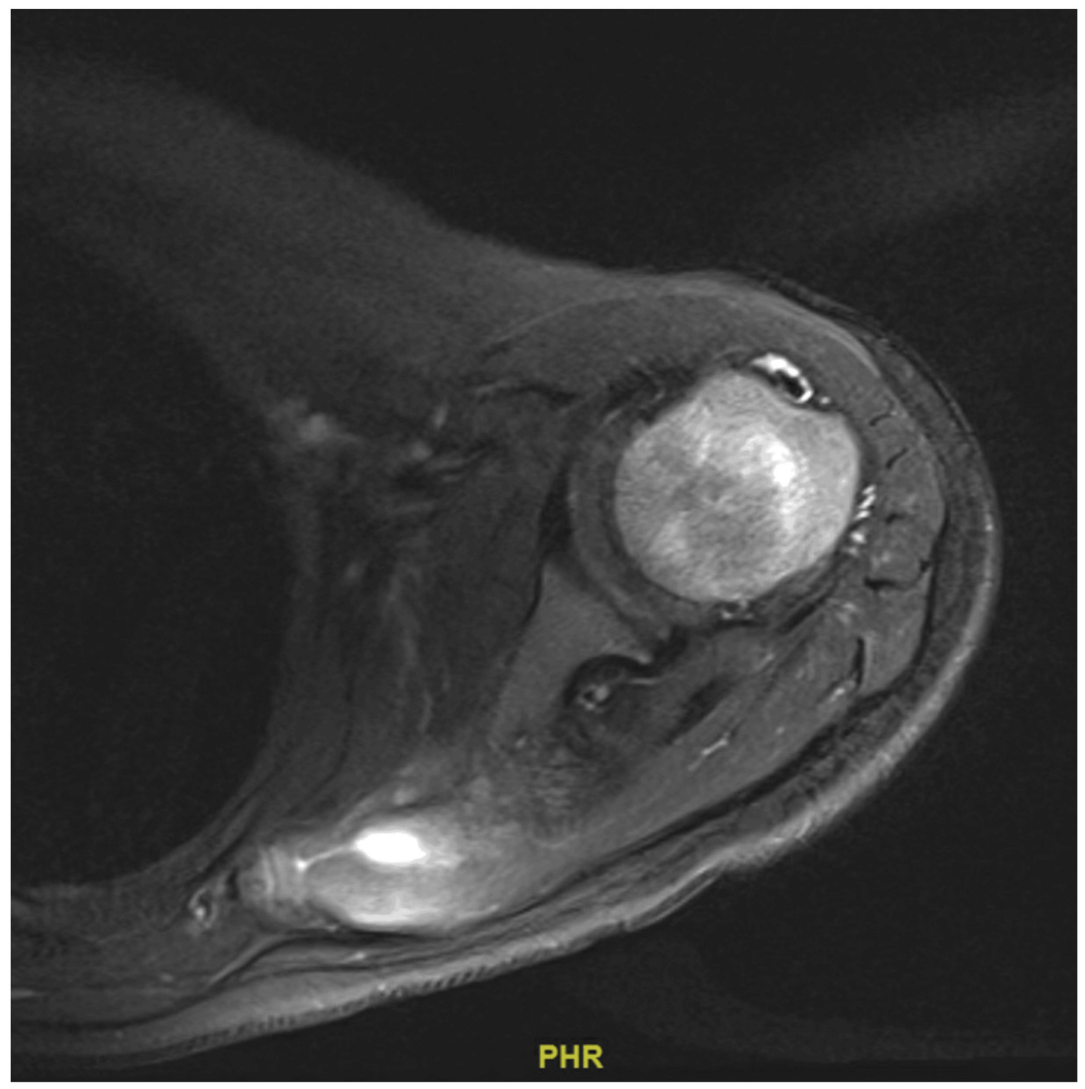
Post-chemotherapy magnetic resonance imaging scan showing residual lesion along the medial aspect of the mid left scapula

After confirming the diagnosis approximately one month after initial presentation, multiple cycles of chemotherapy were initiated and completed within three months. He received a total of four cycles of vincristine, doxorubicin, cyclophosphamide alternating with ifosfamide and etoposide (VDC/IE), which he tolerated well. One month after the patient completed chemotherapy, a CT chest scan with intravenous (IV) contrast and MDP bone scintigraphy were carried out, demonstrating significant interval regression in the size and appearance of the lesion with no signs of lung metastasis (Figures 5-6). MRI with IV contrast demonstrated a lesion along the medial aspect of the left scapula measuring 3 cm x 2.6 cm, with a central necrotic component and a peripheral soft-tissue enhancing component (Figure 7). Finally, wide resection and soft-tissue reconstruction of the left scapula Ewing sarcoma was done successfully without any complications. The patient was discharged on the second postoperative day. 

A follow-up for the patient was done two weeks after the surgery for postoperative assessment. The patient presented in good health, and he then began chemotherapy as part of adjuvant therapy (postop treatment), which was 13 cycles of VDC/IE. At one year postoperatively, a follow-up MRI was done, which showed stable changes that were unlikely to be signs of recurrent disease. A CT scan was performed 18 months postoperatively, which showed residuals of remodeling, sclerosis, and focal small lucencies seen at the body of the left scapula along with adjacent thickening of the pleura and adjacent chest wall. There was no evidence of bone destruction or new soft-tissue enhancing mass. Biopsy was repeated, which confirmed that no recurrence/changes were observed.

## Discussion

Ewing sarcoma presents as an aggressive malignant bone tumor comprising small, round, blue cells, which mainly presents during the first two decades of life, with male predominance [9]. It most commonly affects the long bones of the body and rarely develops in the skull or scapula [1]. The presented case is that of a 10-year-old boy who was found to have Ewing sarcoma in a rare site, the scapula. As per a retrospective study done by Shahbaz Malik et al. involving Ewing sarcoma cases from 1988 to 2018, out of 982 cases, only 29 arose from the scapula [11]. As for symptoms, our patient only complained of painless swelling at the back of his left shoulder, with no signs of infection or any neurological symptoms, and no complaints of affected range of motion. These findings are similar to those of other case reports, which presented with swelling over the shoulder with no restricted range of motion and no reported neurological deficits [5,7]; however, unlike our case, the swelling in those cases was painful. In contrast, other cases were reported to have marked restriction of the shoulder’s range of motion [1,9].

Laboratory studies done for our case included CBC, chemistry, bone panel, liver profile, lactate dehydrogenase (LDH), C-reactive protein, and coagulation profile; all were unremarkable, aside from a hypocalcemia of 1.99 mmol/L, an elevated LDH of 537 U/L, and a slightly elevated coagulation profile. As noted in the literature, anemia, leukocytosis, and elevated erythrocyte sedimentation rate can often be seen in similar Ewing sarcoma cases [12]. As for radiological studies, shoulder US and X-ray were done initially, followed by CT CAP and bone scintigraphy, which showed a large, heterogenous, destructive mass with calcified matrix and no evidence of metastasis. Subsequently, the lesion was biopsied, which found small, round, blue cells that were positive for CD99 and synaptophysin. These uniform, small, round cells that stain positive for CD99 (i.e., the product of MIC-2 genes) are the typical and classical microscopic description for Ewing sarcoma, as they are found in more than 90% of cases [9,12]. Although the presented case was negative for S100, TdT, CD20, desmin, SMA, CK, chromogranin, CD3, and EMA upon immunohistochemistry study, S100 protein and vimentin positivity are commonly observed in such cases [13]. For example, another article reported a case of Ewing sarcoma of the scapula that was positive for CD99, vimentin, and bcl-2 [14].

The presented case received and completed multiple cycles of chemotherapy within two months, which was immediately followed by CT with IV contrast and bone scintigraphy showing regression in the size of the lesion with no metastasis. Finally, the patient underwent wide resection with soft-tissue reconstruction of the left scapula Ewing sarcoma under general anesthesia, which was well-tolerated with no complications. According to the literature, neoadjuvant chemotherapy followed by surgery has been found to be the best management plan for Ewing sarcoma of the scapula in terms of total survival [11]. The prognosis of Ewing sarcoma depends on multiple factors, including anatomical location, tumor size, presence or absence of metastasis, age older than 15 years, and elevated LDH levels [15].

## Conclusions

Ewing sarcoma is the second most common malignant bone tumor in children and adolescents. It usually develops in long bones of the body but can develop in other sites such as the scapula in extremely rare cases. In this article, we presented a case of Ewing sarcoma of the scapula that was confirmed histopathologically and was treated by chemotherapy and surgical resection. At two-year follow-up, a biopsy confirmed the absence of recurrence changes.

## References

[1] Shashaa MN, Hamza A, AlHashemi M, Alyousfi R, Deebo MA, Katnaji J (2021). A child with Ewing's sarcoma in scapula: a rare case report. Int J Surg Case Rep.

[2] Balamuth NJ, Womer RB (2010). Ewing’s sarcoma. Lancet Oncol.

[3] Maheshwari AV, Cheng EY (2010). Ewing sarcoma family of tumors. J Am Acad Orthop Surg.

[4] Burchill SA (2003). Ewing’s sarcoma: diagnostic, prognostic, and therapeutic implications of molecular abnormalities. J Clin Pathol.

[5] Agrawal AC, Yadav SK, Ojha MM, Rakshit J (2020). Giant Ewing's sarcoma of the body of the scapula. J Orthop Case Rep.

[6] Baena-Ocampo Ldel C, Ramirez-Perez E, Linares-Gonzalez LM, Delgado-Chavez R (2009). Epidemiology of bone tumors in Mexico City: retrospective clinicopathologic study of 566 patients at a referral institution. Ann Diagn Pathol.

[7] Waqar SH, Zahid MA (2011). Ewing's sarcoma in scapular region. APSP J Case Rep.

[8] Puchner SE, Panotopoulos J, Puchner R, Schuh R, Windhager R, Funovics PT (2014). Primary malignant tumours of the scapula-a review of 29 cases. Int Orthop.

[9] Biswas R, Krishnan B, Phulware RH (2019). Ewing's sarcoma of scapula: a rare case report. Indian J Surg Oncol.

[10] Ozaki T (2015). Diagnosis and treatment of Ewing sarcoma of the bone: a review article. J Orthop Sci.

[11] Malik SS, Tahir M, Ahmed U, Evans S, Jeys L, Abudu S (2020). Outcome of Ewing's sarcoma of the scapula-a long-term follow-up study. Orthop Traumatol Surg Res.

[12] Shahid M, Varshney M, Maheshwari V, Mubeen A, Siddiqui MA, Julfiqar J, Gaur K (2011). Ewing's sarcoma of scapula: a rare entity. BMJ Case Rep.

[13] Olsen SH, Thomas DG, Lucas DR (2006). Cluster analysis of immunohistochemical profiles in synovial sarcoma, malignant peripheral nerve sheath tumor, and Ewing sarcoma. Mod Pathol.

[14] Bedük-Esen ÇS, Gültekin M, Aydın GB (2019). Ewing sarcoma in an infant and review of the literature. Turk J Pediatr.

[15] Picci P, Böhling T, Bacci G (1997). Chemotherapy-induced tumor necrosis as a prognostic factor in localized Ewing's sarcoma of the extremities. J Clin Oncol.

